# Multiple primary food allergy in pediatric patients seen in a tertiary referral center

**DOI:** 10.1186/2045-7022-1-S1-P34

**Published:** 2011-08-12

**Authors:** Liliane De Swert, Jasmine Leus, Marc Raes, Dominique Bullens

**Affiliations:** 1 University Hospital Gasthuisberg, Pediatrics, Belgium; 2 University Hospital Gasthuisberg, Leuven, Belgium

## Background

Multiple food allergy is increasing.

## Objective

To find out the frequency of multiple primary food allergy (MPFA) in our patient population; the causal foods and their number per patient; age at diagnosis of food allergy; symptoms; frequency of environmental allergy occurring in those patients.

## Methods

MPFA was defined as primary allergy simultaneously occurring to at least 3 unrelated foods. Allergy to a specific food was considered proven based on at least one of the following: a clear cut clinical history; elimination and reintroduction; a specific IgE level or skin prick test size ≥ the 95% positive predictive value (defined for cow's milk, egg, peanut, wheat, potato); a positive provocation test. Primary allergy to the given food was documented by the involvement of class 1 allergens and/or the timing of food allergy development. Files of all patients visiting our tertiary Pediatric Allergy Center between September 2008 and August 2009 were studied retrospectively.

## Results

Out of 715 patients 65 subjects (40 boys/25 girls) were found to have MPFA (9%). Age at enrollment ranged from 6 to 192 months (median 5.8 months); age at first diagnosis ranged from 1 to 192 months (median 11 months; mean 18,5 months). The causal allergens were egg (60/65), cow’s milk (57/65), potato (22/65), peanut (21/65), tree nuts (18/65), fish (15/65), soy (14/65), wheat (10/65), banana (6/65) and kiwi (5/65). In 32/65 subjects more than 3 different foods were involved. The symptoms were: atopic dermatitis, gatrointestinal manifestations, urticaria, angioedema, respiratory manifestations, anaphylaxis and failure to thrive in 92%, 61%, 45 %, 41%, 35 % 28% and 21% respectively. Thirty out of 32 subjects having reached the age of 6 years (93 %) also suffered from respiratory allergy.

## Conclusion

Up to 10% of children visiting a tertiary allergy center might present with MPFA, needing specialized dietary advice.

